# Vibration Sensing Systems Based on Poly(Vinylidene Fluoride) and Microwave-Assisted Synthesized ZnO Star-Like Particles with Controllable Structural and Physical Properties

**DOI:** 10.3390/nano10122345

**Published:** 2020-11-26

**Authors:** Mariem M. Chamakh, Miroslav Mrlík, Stephen Leadenham, Pavel Bažant, Josef Osička, Mariam Al Ali AlMaadeed, Alper Erturk, Ivo Kuřitka

**Affiliations:** 1 Center for Advanced Materials, Qatar University, Doha 2713, Qatar; mariem.chamakh@qu.edu.qa (M.M.C.); m.alali@qu.edu.qa (M.A.A.A.); 2 Centre of Polymer Systems, Tomas Bata University in Zlin, Trida T. Bati 5678, 760 01 Zlin, Czech Republic; bazant@utb.cz (P.B.); osicka@utb.cz (J.O.); kuritka@utb.cz (I.K.); 3 G. W. Woodruff School of Mechanical Engineering, Georgia Institute of Technology, Atlanta, GA 30332, USA; leadenham1@llnl.gov (S.L.); alper.erturk@me.gatech.edu (A.E.)

**Keywords:** poly(vinylidene fluoride), ZnO star-like particles, structure characterization, physical properties, vibration sensing

## Abstract

This study deals with the effect of zinc oxide (ZnO) star-like filler addition to the poly(vinylidene fluoride) (PVDF) matrix, and its effect on the structural and physical properties and consequences to the vibration sensing performance. Microwave-assisted synthesis in open vessel setup was optimized for the preparation of the star-like shape of ZnO crystalline particles. The crystalline and star-like structure was confirmed by X-ray diffraction (XRD), scanning electron microscopy (SEM) and energy-dispersive spectroscopy (EDX). Furthermore, the PVDF-based composites were prepared using a spin-coating technique from solution. An investigation of the transformation of the *α* crystalline phase to the *β* crystalline phase of the neat PVDF matrix and with various filler concentrations was performed using Fourier-Transform infrared (FTIR) spectroscopy, which shows an enhanced *β*-phase from 44.1% to 66.4% for neat PVDF and PVDF with 10 wt.% of particles, respectively. Differential scanning calorimetry (DSC) measurements and investigation showed enhanced crystallinity and melting enthalpy of the composite systems in comparison to neat PVDF, since ZnO star-like particles act as nucleating agents. The impact of the filler content on the physical properties, such as thermal and dynamic mechanical properties, which are critical for the intended applications, were investigated as well, and showed that fabricated composites exhibit enhanced thermal stability. Because of its dynamic mechanical properties, the composites can still be utilized as flexible sensors. Finally, the vibration sensing capability was systematically investigated, and it was shown that the addition of ZnO star-like filler enhanced the value of the thickness mode *d*_33_ piezoelectric constant from 16.3 pC/N to 29.2 pC/N for neat PVDF and PVDF with 10 wt.% of ZnO star-like particles.

## 1. Introduction

Poly(vinylidene fluoride) (PVDF) is a semicrystalline polymer that shows unusual behavior, exhibiting excellent processing in the form of films and physical properties, as well as tailorable structural and electro-active capabilities [1,2,3,4]. These capabilities promote the applicability of this polymer in different systems (i.e., controllable actuation, sensing and energy harvesting [5,6,7,8]).

PVDF has an extraordinary semicrystalline origin, and especially the crystalline phase is crucial for the aforementioned applications. The electrically active *β*-phase is one of the three crystalline phases that mainly occurs in a PVDF polymer. In case of an *α*-phase polymer, backbones are in alternating directions and show a paraelectric performance; therefore, a non-polar type of polymer is present. Nevertheless, the *β*- and *γ*-phases and the significantly suppressed non-polar *α*-phase provide polymer with prevailing polar behavior [9,10,11].

Generally, PVDFs form the *α* crystalline phase which is in many cases without specific applications, due to the aforementioned reasons. However, there are various method to enhance the *β*-phase content in the PVDF material for already-fabricated film. Film stretching is a very promising approach, as published elsewhere [12,13]. Moreover, the poling of the film uses a high electric field strength in the order of several kV mm^−1^ [14]. Interestingly, the application of these two methods together exhibit an enhanced *β*-phase development in comparison to individual methods alone [15]. A further possibility for *β*-phase improvement is employing various PVDF film preparations via electrospinning [16,17,18,19] or using blending procedure with some polymers, i.e., poly(methyl methacrylate) [20]. The fabrication of the PVDF films with a certain number of particles as a filler can also be used for *β*-phase improvement. These fillers can be organics (graphene oxide, reduced graphene or graphite) [2,21,22] or ceramic (BaTiO3, PbTiZrO_5_) [23,24], metals and metal oxides (Cu, Co, Ag, ZnO, CoFe_2_O_4_ or TiO_2_) [25,26,27,28,29,30] or as hybrid systems based on ZnO/GO [31]. ZnO particles of various shapes were already utilized to improve the *β*-phase, such as in ZnO nanoparticles [32] and ZnO nanowires [33]. Surprisingly, the fabrication of the Hydrangea-like structures was not able to develop a *β*-phase in the PVDF system, although the dielectric properties were considerably enhanced; it seems that 10–60 wt.% reflects very high loading to provide sufficient nucleation and transformation of the crystalline phase. [34].

The preparation of the fillers based on TiO_2_, ZnO or Ag of various shapes by employing conventional hydro or solvo-thermal procedures, which are high temperature, pressure and time demanding processes, is considerably cost-ineffective [35,36,37]. In recent decades, there have been different microwave-assisted procedures able to provide particles of an appropriate shape and structure that have sufficiently low costs [38,39,40,41]. Effectivity and short reaction times are some of the benefits that can shift this approach toward large-scale production.

In order to provide system possessing-enhanced sensing capability to vibrations, the *β*-phase needs to be well-developed, as is stated in the following references [42,43]. In this case, the vibration sensor is connected to the piezoelectric activity, showing that if piezoelectric coefficients will be enhanced, it is mostly due to the well-developed electro-active *β*-phase. Additionally, the vibration sensor provides higher values by means of sensitivity, which was detected on a real electrical device [44].

The current study was mainly focused on the preparation of microwave-assisted ZnO star-like particles and understanding their influence on the physical properties (calorimetric, thermal and mechanical), and how the *β*-phase development is affected by this type of filler in the PVDF-based composite films. Moreover, it was elucidated how the change in *β*-phase content and change of other physical properties influence the applications involving vibration sensing during mechanical excitations under various electric loads.

## 2. Materials and Methods 

### 2.1. Materials

Zinc acetate dihydrate Zn(CH_3_COO)_2_ · 2H_2_O (ZAD) and aqueous ammonia (25–29 wt.%; NH3 aq.) were purchased from PENTA (Prague, Czech Republic). Acetone 98%, N,N-dimethylformamide 99% (DMF), poly(ethylene glycol) (PEG; molecular weight = 400) and poly(vinilidene fluoride) (PVDF; molecular weight = 275,000) were purchased from Sigma-Aldrich (Prague, Czech Republic). All chemicals were of analytical grade and used as received without further purification. Demineralized (DEMI) water of 25 µS m^−1^ was used in all of these experiments.

### 2.2. Synthesis of ZnO Star-Like Particles

ZnO star-like particles were synthesized using open vessel MWG1K-10 (RaDan, Trinec Czech Republic) operating at 2.45 GHz and 1100 W of power under hydro-thermal conditions using a microwave. Schematic illustration of the open vessel system is presented in Figure S1. In this case, the reactants were mixed with DEMI water in the following procedure: solution I was prepared by dissolving 10.8 g ZAD in 80 mL of water, solution II was prepared by dissolving 14.2 mL of NH_3_ aq. in 40 mL of DEMI water and solution III was prepared by dissolving 5.142 g of PEG in 20 mL of DEMI water. Firstly, solution I and III were mixed together followed by the addition of solution II, while 140 mL was maintained as the final volume of the reaction batch. The mixture was placed into a 250 mL reaction bottle and transferred to a microwave reactor connected to the external cooler. The reaction was started by the application of microwaves and stopped after 10 min. The temperature evolution during the reaction was checked by an external thermometer, and most of the time this was 103 ± 1 °C. The resulting solution was cooled down at ambient conditions. Finally, the whole reaction solution was filtrated using cellulose membranes with 0.44 μm thickness, using DEMI water repeatedly. The resulting material in the form of powder was dried under vacuum overnight.

### 2.3. Preparation of the PVDF/ZnO Composite Films

The neat PVDF and PVDF composite films with various content of ZnO star-like particles were fabricated via the spin-coating technique. Firstly, the corresponding amount of ZnO star-like particles were dispersed in a 2 mL of DMF, mechanically with a glass stick, after which they were ultrasonically agitated for 5 min. Then, 1.4 g of PVDF was added to the ZnO star-like particle solution together with the corresponding amount of additional DMF in order to achieve the same solution concentration for all solutions and stirred at 60 °C until the PVDF was completely dissolved. Next, the solutions containing 0.5, 1, 3, 5 and 10 wt.% of ZnO star-like particles in comparison to PVDF were spin-coated on the square glass substrates (with a dimension of 5 cm × 5 cm) and covered by aluminum foil. A spin-coating process was optimized at a velocity of 2500 rpm and an acceleration of 500 rpm·s^−1^. In all cases, 1 mL of the solution was put on the substrate and the process was performed under nitrogen atmosphere. The rate of dropping was 0.1 mL per 1 min and ambient temperature and pressure were used.

### 2.4. Poling of the Prepared Spin-Coated Films

The neat PVDF as well as the PVDF-based Zn O composite analogues were sputtered with copper from both sides and poled in the thickness (3-3-0 direction) using a high-voltage source (TREK, Lockport, NY, USA). The films have 200 ± 10 μm in thickness and were used in this form for further analyses.

### 2.5. General Characterization

The crystalline phase of the ZnO star-like powders was measured using the X-ray diffractometer X’Pert PRO X-ray (PANalytical, Almelo, The Netherlands) with a Cu-K*_α_* X-ray source (λ = 1.5418 Å) in the range from 5° to 85°. The same powders were analyzed using Fourier-Transform infrared (FTIR) 6700 Nicolet (Thermo Scientific, USA) in ATR mode in a wavenumber range of 4000–500 cm^−1^ in order to investigate the successful composite preparation and investigation of the *β*-phase development. The star-like shape of the ZnO samples was measured using Scanning electron microscopy (SEM) together with energy-dispersive spectroscopy (EDX) (Tescan, Brno, Czech Republic), which provides the elemental analysis of the ZnO star-like samples.

Thermal properties were performed using a thermo-gravimetric analysis (TGA) on Perkin Elmer Pyris 6 TGA (Perkin Elmer, Billerica, MA USA), ranging from 50 °C to 800 °C under inert atmosphere and a heating rate of 10 K/min. In order to investigate the calorimetric properties of the investigated samples, differential scanning calorimetry (DSC) was used, namely a Perkin Elmer model DSC 8500 (Perkin Elmer, Billerica, MA, USA). The PVDF films with a weight in the range of 2.2–4.5 mg were measured in two heating/cooling cycles in a temperature range of 100–220 °C. For the final evaluation the first cycle was used and calculated with the Perkin Elmer Pyris software 6.71b, Akron, OH, USA).

A dynamic mechanical analysis (DMA) in tensile mode was performed by an RSA-G2 (TA Instruments, New Castle, DE, USA). The PVDF films were cut using a stainless steel template and have a dimension of 35 mm × 4 mm × 0.2 mm (length × width × thickness). The dynamic mechanical properties were investigated in the linear viscoelastic region (0.05% strain deformation) in a temperature range from −100 °C to 100 °C at a frequency of 1 Hz and under inert atmosphere.

### 2.6. Vibration Sensing under Mechanical Excitation

**Figure 1:**
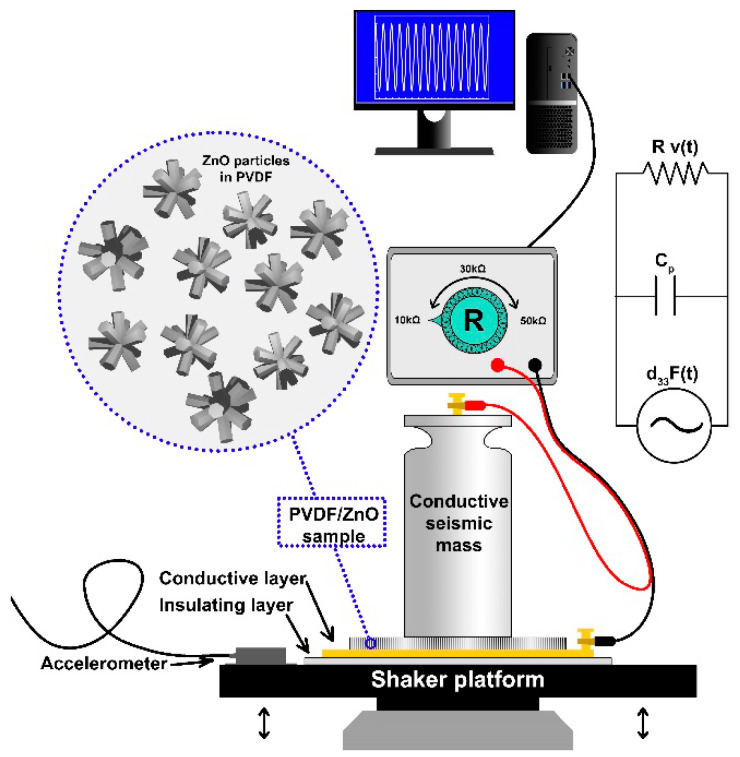
Schematic of the experimental setup to identify the thickness mode piezoelectric constant and lumped parameter circuit diagram of the sample.

To identify the thickness mode piezoelectric constant of each sample, a series of dynamic experiments were conducted. As schematically shown in Figure 1, the setup consists of the PVDF sample sandwiched between a conductive seismic mass on top and a conductive foil layer below. The seismic mass and the foil layer form the electrodes, with wires connecting them to the shunt resistance box. Beneath the foil layer is an insulating layer that isolates the sample from the metal shaker platform, which oscillates harmonically in the vertical direction. An accelerometer is used to measure the motion of the shaker table.

A lumped parameter model of a piezoelectric device in thickness (3-3 mode) shunted by a load resistance can be obtained as [45]:

(1) $$\dot{F}d_{33}=C\dot{v}+\frac{v}{R}$$

where *F* is the force on the test sample in the poling direction, *d*_33_ is the thickness mode piezoelectric constant, *C* is the static capacitance of the test sample, *v* is the voltage across the electrodes, *R* is the load resistance and the overdot represents the derivative with respect to time. For these tests, the dynamic forcing is provided by the shaker motion and the seismic mass. That is:

(2) F(t)=ma(t)

where *m* is the mass of the seismic mass and *a*(*t*) is the vertical acceleration of the shaker table. This model is applicable when the frequency of the applied force is well below the fundamental resonance frequency of the system formed by the elastic PP/PVDF sample and the seismic mass. In the tests done here, the forcing frequencies are around the 10–100 Hz range, while the resonant frequency of the system is well above 10 kHz. Under harmonic excitation, using Equations (1) and (2) one can obtain the following complex frequency response function, which relates the electrode voltage output to the base acceleration input:

(3) $$\frac{V}{A}=\frac{jRmd_{33}\Omega }{jRC\Omega +1}$$

where *j* is the unit imaginary number and Ω is the excitation frequency in rad s^−1^. To extract the *d*_33_, of each sample, harmonic excitation frequency sweep tests were conducted with various load resistances. Mass values were chosen to isolate the frequencies of interest for the tests from spurious vibration modes in the shaker and shaker table. All masses have the same contact area of 9.56 cm^2^. Frequencies chosen were low (*RC*Ω << 1), yielding experimental frequency response functions that have an approximately linear dependence on the excitation frequency:

(4) $$\left|\frac{V}{A}\right|\approx Rmd_{33}\Omega$$

The abovementioned measuring procedure was already used for the investigation of the piezoelectric constant elsewhere [46].

## 3. Results and Discussion

### 3.1. Synthesis of ZnO Star-Like Particles

**Figure 2:**
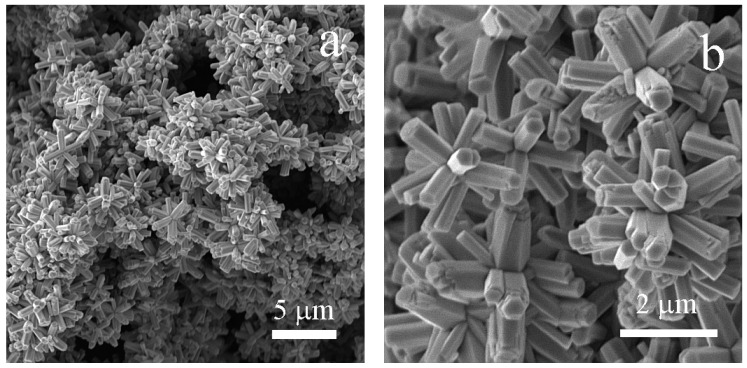
Scanning electron microscopy (SEM) images ZnO star-like particles under various magnifications: (**a**) 5000× and (**b**) 20,000×.

The previously described approach in the experimental section results in well-developed three-dimensional (3D) star-like superstructures (Figure 2). It has to be noted that the ZnO can also form very similar structures, and in the literature, “flower-like” shape can be also found for these types of ZnO particles [47]. As can be seen in Figure 2a, the star-like shape was developed for a large number of particles and the particle size distribution seems to be very narrow. This is also confirmed in Figure 2b, where the star-like particles are under higher magnification; it can be clearly observed that their mean size is 2.5 ± 0.3 μm in diameter and the mean length/thickness ratio of the microrod forming the star-like shape is 2 ± 0.6. Therefore, it can be stated that the microwave-assisted synthesis provides a well-developed star-like shape with narrow particle size distribution, which is required for our potential application.

**Figure 3:**
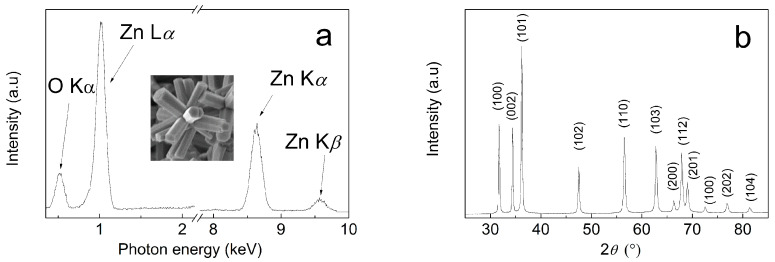
Energy dispersive spectrum (**a**) and X-ray diffraction spectrum (**b**) of ZnO star-like particles. Inset of the Figure 2a is the area from which the energy-dispersive spectroscopy (EDX) was collected.

In order to confirm the purity of the particles and presence of the ZnO structures, EDX and XRD measurement were performed (Figure 3). As can be seen from the EDX investigations (Figure 3a), the star-like particles, including expected elements such as zinc and oxygen, are clearly present. At low energies it can be seen that the L*_α_* shows an emission peak for zinc and *K_α_* shows a peak for oxygen. As also observed frequently by other researchers [38,48], the emission peaks of *K_α_* and *K_β_* for zinc usually appear at high energies. However, other peaks for further elements were not observed, indicating the high purity of the samples. Similar spectra were obtained for conventional ZnO particles (Figure S2).

Further confirmation of the high purity of the ZnO star-like particles were given by XRD investigations (Figure 3b). Here, the diffraction peaks for relative intensities are observed at 2θ = 31.7°, 34.4°, 36.2° 47.5°, 56.6°, 62.8°, 67.8°, 68.9°, 72.47° and 81.35°, which match perfectly with the ZnO hexagonal wurtzite crystal structure according to the JCDD PDF-2 entry 01-079-0207. This is deemed a sufficient validation of the high purity and ZnO crystalline structure of the synthesized star-like particles.

### 3.2. Structural Characterization of the ZnO Star-Like/PVDF Composite Films

**Figure 4:**
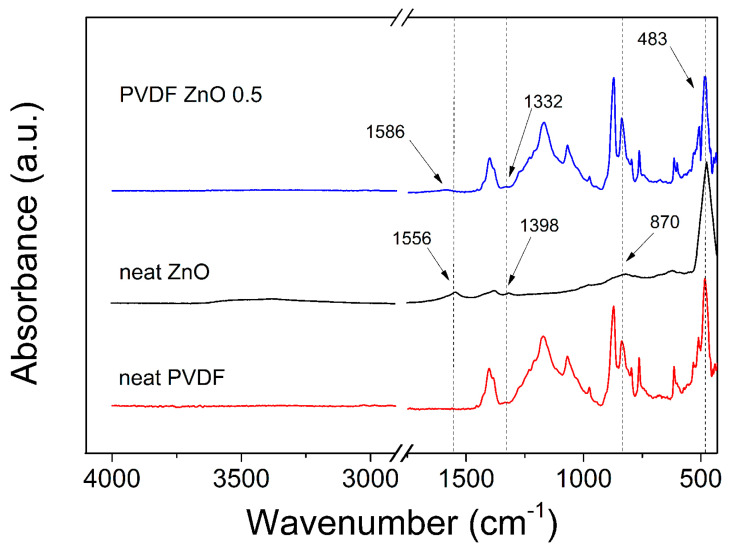
Fourier-Transform infrared (FTIR) spectra for ZnO particles (black) and spin-coated films of neat PVDF (red) and PVDF/ZnO 0.5 composite (blue).

In order to investigate the structural properties of the prepared spin-coated films, an FTIR measurement was performed. In Figure 4, the neat PVDF, the neat ZnO star-like particles and the composite film including 0.5 wt.% of the ZnO particles are shown. Here, it can be seen that neat ZnO particles include a small amount of water, which is confirmed by a very broad peak between 3500 cm^−1^ and 3000 cm^−1^ as well as between 1556 cm^−1^ and 1398 cm^−1^. The peaks below 1000 cm^−1^ are specific peaks for the Zn–O structure, especially the peak at 483 cm^−1^. The neat PVDF matrix has specific FTIR spectra, which will be discussed below. The composite film, including the PVDF matrix and 0.5 wt.% of ZnO star-like filler, reflects the peaks from all individual components. In order to clearly confirm the successful film preparation, an FTIR spectrum with the lowest filler content was chosen. Here, it can be expected that if the characteristics peaks of ZnO are made visible, for higher filler contents, the peaks can be observed even more explicitly. However, the FTIR results will be discussed from an electro-activity point of view in more detail later.

**Figure 5:**
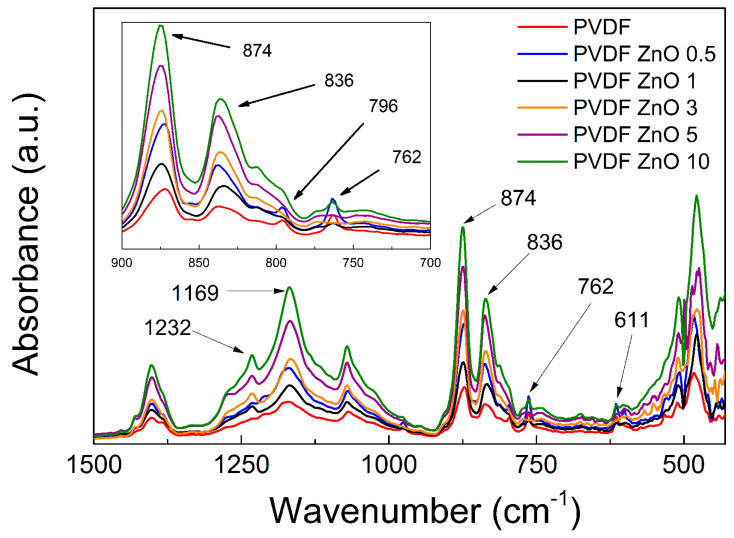
FTIR spectra for neat PVDF and the PVDF ZnO star-like composite films with various filler contents.

The FTIR spectra measured from 450 cm^−1^ up to 1500 cm^−1^ (Figure 5) was used for the investigation of the transformation between the a-phase and b-phase crystalline phase. Specific peaks for the *α*-phase are present at 485, 611, 762,796 and 975 cm^−1^; these band were clearly determined elsewhere at similar wavenumbers [24,27,35]. The most important peaks for the *α*-phase are 611 and 762 cm^−1^. The peaks observed at 836, 874, 1169 and 1232 cm^−1^ correspond to the *β*-phase [27,32]. Following absorption at 878 cm^−1^ represent the CH_2_. The CF_2_ absorption is connected to the stretching and is visible at 1171 cm^−1^, while wagging of the CH_2_ group is present at 1232 cm^−1^. The most important peaks relevant to the improved *β*-phase and suppressed *α*-phase are present at 836 cm^−1^ and 762 cm^−1^, respectively. In order to calculate the relative ratio between these two phases, the ratio of the peak intensities can be seen in the Table 1 for the neat PVDF film as well as for the composite films with various content of ZnO star-like particles. As can be seen in Table 1, the ratio is 1.9 and is the same for neat PVDF, as reported elsewhere [26], while with an increasing number of the ZnO star-like particles, this ratio increases and reaches the highest value of 22.5 for composites with 10 wt.% filler content confirming the well-developed *β* crystalline phase.

Moreover, the *β*-phase investigation according to the following Equation (5) is also frequently used [49,50,51]:

(5) $$F\left(\beta \right)=\frac{A_{\beta }}{\frac{\kappa_{\beta }}{\kappa_{\beta }}A_{\alpha }+A_{\beta }}$$

where *A_α_* and *A_β_* are values of absorbance corresponding to the wavenumber 762 cm^−1^ and 840 cm^−1^, respectively, and *κ_α_* and *κ_β_* are absorption coefficients for the *α*-crystalline phase and *β*-crystalline phase, having values 6.1 × 10^4^ cm^2^ mol^−1^ and 7.7 × 10^4^ cm^2^ mol^−1^, respectively [52]. The results from Equation (5) are summarized in Table 1.

The amount of *β*-phase for neat PVDF (44.1%) is very similar to those observed in other publications [51,52]. Further addition of ZnO star-like particles into the PVDF matrix gradually improves the *β*-phase development. Small addition (0.5 and 1 wt.%) just negligibly affects the final value, while the samples PVDF ZnO 5 and PVDF ZnO 10 exhibit improved values of 60.9% and 66.4%, respectively. These values are not as high as the values observed for electrospun fiber mats [49,50], but are still very promising for the samples intended to be applied as vibration sensors. This transformation from *α*-phase to *β*-phase is mainly caused due to the presence of particles that are suppressing the movement of the fluorine atoms along the main backbone, thus providing a more ordered formation that is mostly connected to the *β*-phase.

**Figure 6:**
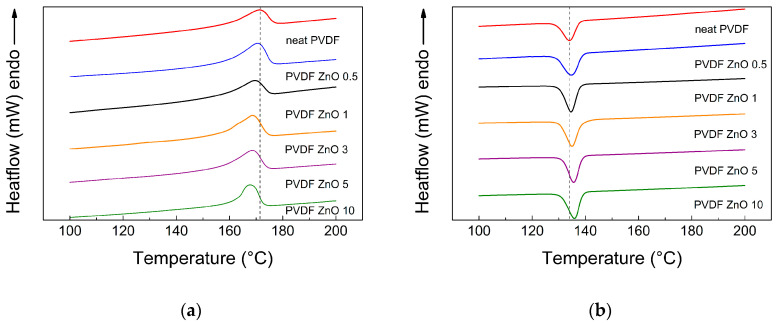
Differential scanning calorimetry (DSC) curves for melting (**a**) and crystallization (**b**) of neat PVDF and PVDF ZnO star-like composite films with various filler contents.

For a deeper structural investigation of the prepared samples from a melting and crystallization point of view, DSC measurements were performed and the first cycle was evaluated (Figure 6). As can be clearly seen from Figure 6a, the melting temperature, *T*_m_, decreases with increasing filler content and the highest difference for neat PVDF and PVDF ZnO 10 is 4 °C (Table 2). This significant decrease can be attributed to two main reasons. The first is connected to the fact that ZnO star-like particles act as nucleation agents, disturb the possible crystal perfection and contribute to the formation of the large number of crystallites with a small size, leading to easier melting. The second reason concerns the presence of the ZnO star-like particle increase in thermal conductivity; thus, the applied heat is better distributed within the sample, leading to melting at lower temperature of the crystalline structure [34]. On the other hand, the melting enthalpy, Δ*H*_m_, of the crystalline phase slightly increases and provide more uniform crystallites, resulting in a narrower peak distribution of the melting peak. In case of the crystallization process, as the filler content increases, the crystallization temperature, *T*_c_, increases as well (Figure 6b). This behavior is connected to the higher interface energy, which strongly influences the *T*_c_, where higher interface energy increases *T*_c_ due to the increasing amount of the ZnO star-like filler in the samples. Similarly, the crystallization enthalpy, Δ*H*_c_, increases with increasing filler content as a result of the crystallization nucleation caused by the presence of the ZnO star-like particles in the samples. All values for crystallization and melting processes are summarized in Table 2.

In order to confirm that the presence of the ZnO star-like particles positively affect the amount of crystalline phase, the crystallinity, *X*_c_, of the PVDF based samples was calculated according to Equation (6) [53] and summarized in Table 2.

(6) $$X\left(c\right)=\frac{\Delta H_{m}}{\Delta H_{m}^{0}}\times 100$$

where Δ*H*_m_ is the heat of fusion for individual samples and $\Delta H_{m}^{0}$ is the heat of fusion obtained for 100% crystalline PVDF (104.5 J g^−1^).

It can be concluded that the amount of the ZnO star-like particles in the PVDF matrix influence the overall crystallinity, where it gradually increases with increasing filler loading. If the overall crystallinity increases, the transformation of the *α*-phase to the electro-active *β*-phase can also be easily achieved by simple polling, as was used in our case.

#### Thermal Properties of the ZnO Star-Like/PVDF Composite Films

**Figure 7:**
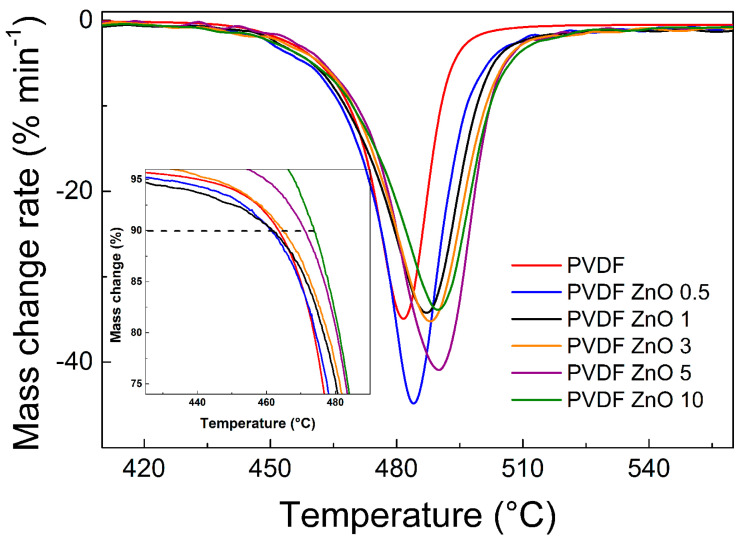
Thermogravimetric spectra in its derivation form for neat PVDF and the PVDF ZnO star-like composite films with various filler contents. Inset figure is highlighted the *T*_10%_ evaluation.

The thermal properties of the prepared composite films were investigated by TGA analysis. As seen in Figure 7, the peak (assigned as *T*_max_) observed at the mass change rate, which is derived from the typical TGA experimental data, shows the lowest value of 481.54 °C for the neat PVDF film and it is increasing with an increasing amount of ZnO star-like particles, which are able to shift the resistance against the thermal exposure to the higher temperatures: 489.90 °C for PVDF ZnO 10 composite film. Similar behavior was observed in other studies where ZnO-based particles were used [34]. In Figure 7, interestingly, it can be seen that there is an improvement of the thermal properties (*T*_10%_) that is not present from the beginning, especially in the case of low particle contents up to 3 wt.%. Only the largest amount of particles addition provides the thermal stability improvement in the whole investigated temperature range. Finally, all values investigated within the TGA are summarized in Table 3.

### 3.3. Dynamic Mechanical Properties of the ZnO Star-Like/PVDF Composite Films

**Figure 8:**
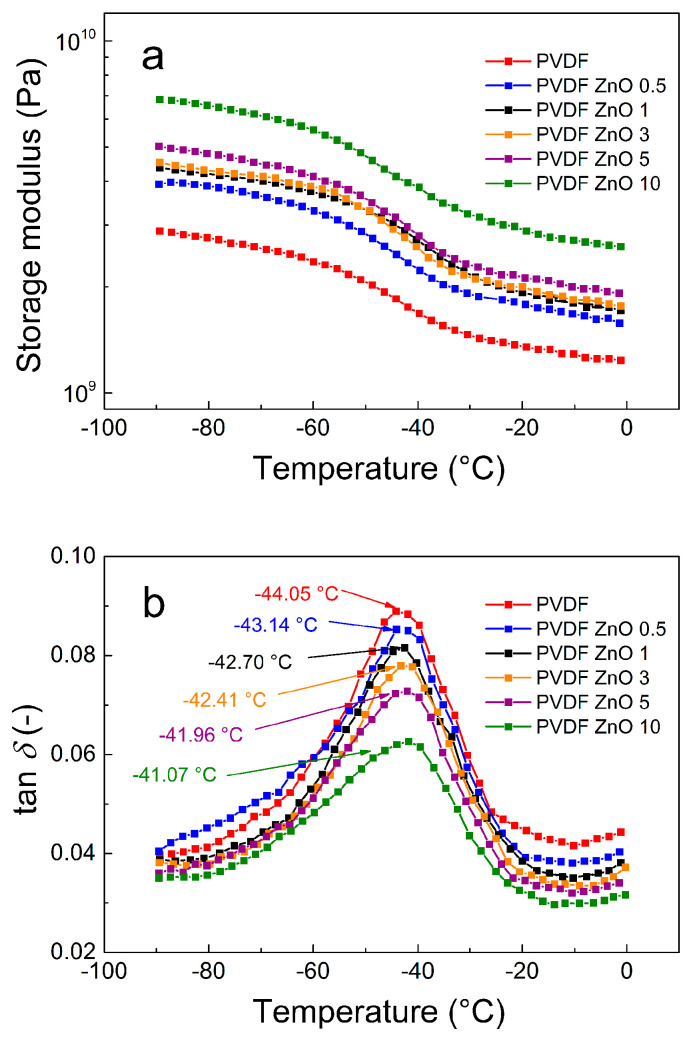
Temperature dependence of the storage modulus (**a**) and tan δ (**b**) for neat PVDF and the PVDF ZnO star-like composite films with various filler contents.

In order to investigate how PVDF films were changed structurally by ZnO star-like filler addition, the DMA curves were analyzed with respect to the increasing temperature profiles (Figure 8). As seen in Figure 8a, the storage modulus reflecting the elastic portion of the material increases with an increasing amount of the filler content, while it decreases with an increasing temperature. However, the decreasing rate of the storage modulus is the smallest for the PVDF ZnO 10 sample, indicating that the ZnO star-like particles strengthen the polymer films. These findings were also confirmed by increasing the glass transition temperature (*T*_g_), which indicates that the PVDF polymer chain suffers from the presence of the ZnO star-like particles (Figure 8b), when the *T*_g_ of the neat PVDF matrix and for PVDF ZnO 10 are −44.05 °C and −41.07 °C, respectively. Additionally, the peak of the *T*_g_ decreases with increasing filler content, indicating that good interactions between the filler and matrix was achieved, causing a significant improvement of the mechanical properties.

**Figure 9:**
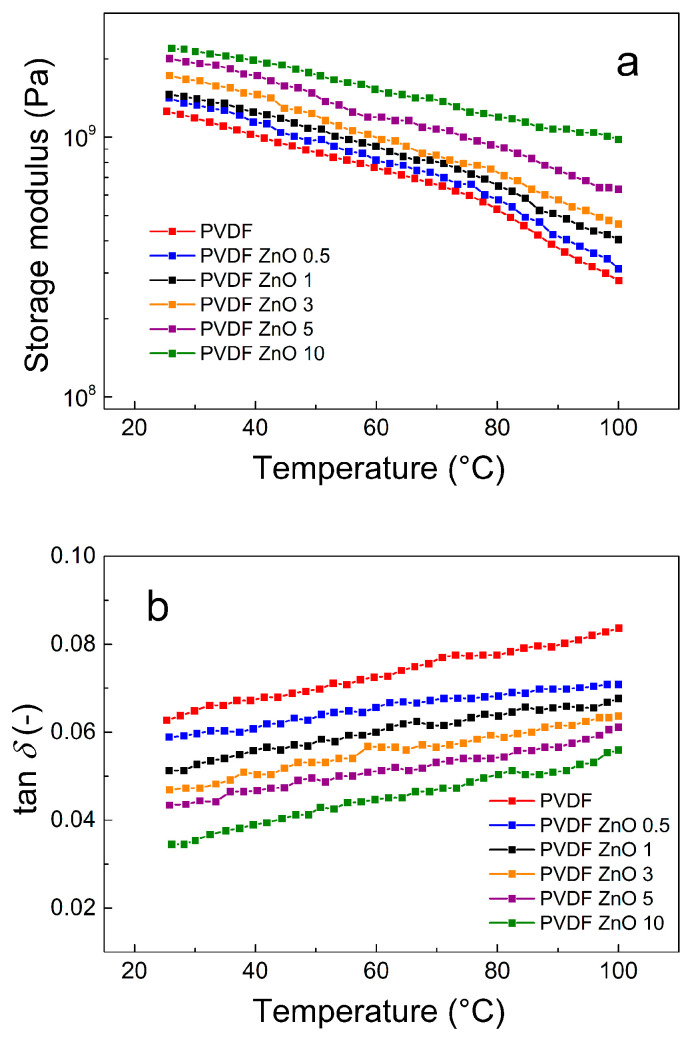
Temperature dependence of the storage modulus (**a**) and tan δ (**b**) for neat PVDF and the PVDF ZnO star-like composite films with various filler contents.

Furthermore, the DMA properties of the prepared composites films were measured up to 100 °C, as this temperature range is expected to be the operation window for these composite films (Figure 9). As seen in the Figure 9a, the storage modulus follows a similar pattern in the case of lower temperatures and was measured to above 10^8^ Pa for all films, while in the case of PVDF ZnO 10, composite film reaches 10^9^ Pa at 100 °C, providing suitable mechanical properties for possible multiple applications. Moreover, the values of the tan δ do not exceed a value of 0.1, which clearly indicates that all prepared composites possessed strongly elastic behavior in the whole investigated temperature range (Figure 9b). The PVDF ZnO 10 sample exhibits the lowest values of tan δ, confirming the fact that this sample has the best dynamic mechanical response at a broad temperature range, providing the novel composite material with promising applications.

### 3.4. Vibration Sensing Capability upon Mechanical Excitation

The vibration sensing capability of materials based on PVDF mainly depends on the amount of the *β*-phase developed in the individual samples [14]. As previously mentioned in the introduction, stretching or poling can be employed to improve the *β*-phase crystallites and ensure good performance. In our case, only the poling method was used for this purpose, and a very simple spin-coating approach was utilized for the film preparation, in accordance with the amount of the *β*-phase vibration sensing capability, represented by a *d*_33_ piezoelectric constant, which increases with an increasing amount of ZnO star-like particles (Table 4). The *d*_33_ piezoelectric constant in case of the neat PVDF is 16.3 pC/N, which corresponds well to the values obtained for commercial bench-top PVDF [54]. After the addition of a low number of ZnO particles (PVDF ZnO 0.5 and PVDF ZnO 1), the *d*_33_ increased very slightly to 18.9 pC/N and 19.1 pC/N, while in the higher filler loading the *d*_33_ increased to 23.2 pC/N and 26.2 pC/N for PVDF ZnO 3 and PVDF ZnO 5, respectively. Finally, the best performance was observed for sample PVDF ZnO 10, due to the best-developed *β*-phase in its structure and because it provides a suitable performance that can be applied as vibration sensors for different applications, such as structural health monitoring.

## 4. Conclusions

In this study, a simple approach for polymer composite films preparation based on PVDF and ZnO star-like particles for vibration sensing was developed. A modified microwave-assisted synthesis for the fabrication of the ZnO star-like particles was introduced. The ZnO crystalline structure was confirmed by XRD, while the ZnO purity was proven by EDX spectroscopy. Successful fabrication of the composite films via spin-coating approach was investigated by FTIR. Presence of the ZnO star-like particles induced the *β*-phase crystallites development, elucidated by FTIR and DSC. Thermal properties as well as dynamic mechanical properties were significantly enhanced due to the presence of the ZnO star-like particles. Since vibration sensing is based on mechanical deformation of the sample, DMA investigation also revealed the suitability of such films for the intended applications. The thickness mode piezoelectric constant (*d*_33_) showed 16.3 pC/N for the neat PVDF sample and considerably improved value of 29.2 pC/N for the PVDF sample with 10 wt.% of the ZnO star-like particles. These modified and easily tailored films can find promising application in vibration sensing.

## Figures and Tables

**Table 1:** Summarized values of the relative ratio between the intensities corresponding to *α*- and *β*-phase and the calculation of the *β*-phase according to Equation (5).

Sample Code	Neat PVDF	PVDF ZnO 0.5	PVDF ZnO 1	PVDF ZnO 3	PVDF ZnO 5	PVDF ZnO 10
Ratio	1.9	8.3	8.7	14.4	18.9	22.5
*F* (β)	44.1	46.2	47.3	53.8	60.9	66.4

**Table 2:** Summarized values of the DSC curves investigation for neat PVDF and PVDF ZnO star-like composite films with various filler contents.

Sample Code	*T*_m_ (°C)	Δ*H*_m_ (J g^−1^)	*T*_c_ (°C)	Δ*H*_c_ (J g^−1^)	*X*_c_ (%)
neat PVDF	171.73	41.96	134.09	−51.72	40.2
PVDF ZnO 0.5	170.44	43.16	134.47	−53.95	41.3
PVDF ZnO 1	169.69	44.48	134.65	−54.99	42.6
PVDF ZnO 3	168.77	47.16	135.02	−56.84	45.1
PVDF ZnO 5	168.59	50.45	135.58	−59.42	48.3
PVDF ZnO 10	167.84	52.56	135.76	−61.36	50.3

**Table 3:** Summarized values of the TGA curves investigation for neat PVDF and PVDF ZnO star-like composite films with various filler contents.

Sample Code	*T*_10%_ (°C)	*T*_max_ (°C)
neat PVDF	463.4	481.5
PVDF ZnO 0.5	461.3	483.9
PVDF ZnO 1	461.7	486.9
PVDF ZnO 3	465.3	488.1
PVDF ZnO 5	471.1	489.1
PVDF ZnO 10	473.8	489.9

**Table 4:** Summarized values of *d*_33_ identification.

Sample Name	10 kΩ (pC/N)	30 kΩ (pC/N)	50 kΩ (pC/N)	Avg. (pC/N)
pure PVDF	16.4	16.6	16.01	16.3 ± 0.3
PVDF ZnO 0.5	18.7	19.00	19.1	18.9 ± 0.2
PVDF ZnO 1	18.8	19.4	19.3	19.1 ± 0.3
PVDF ZnO 3	23.2	23.9	22.5	23.2 ± 0.7
PVDF ZnO 5	26.2	25.7	25.5	25.8 ± 0.4
PVDF ZnO 10	28.9	29.3	29.4	29.2 ± 0.3

## Supplementary Materials

The following are available online at https://www.mdpi.com/2079-4991/10/12/2345/s1, Figure S1: Schematic illustration of the microwave (MW) open vessel apparatus where is 1—external MW source, 2—reaction vessel with temperature control, 3—MW oven, 4—defoamer, 5—dropping system, 6—Allihn condenser, Figure S2: Energy dispersive spectrum (a) and X-ray diffraction spectrum (b) of conventional ZnO particles.
