# Supplementary material for: Vibration Sensing Systems Based on Poly(Vinylidene Fluoride) and Microwave-Assisted Synthesized ZnO Star-Like Particles with Controllable Structural and Physical Properties
Source: Nanomaterials (Basel). 2020 Nov 26;10(12):2345. doi: 10.3390/nano10122345 (PMC7761233; doi:10.3390/nano10122345)
Supplement: Supplementary file 1 [file nanomaterials-10-02345-s001.pdf]

nanomaterials

Supporting Information

Vibration Sensing Systems Based on Poly(Vinylidene Fluoride) and Microwave-Assisted Synthesized ZnO Star-Like Particles with Controllable Structural and Physical Properties

Mariem M. Chamakh [1], Miroslav Mrlík [2,*], Stephen Leadenham [3], Pavel Bažant [2], Josef Osička [2], Mariam Al Ali AlMaadeed [1], Alper Erturk [3] and Ivo Kuřitka [2]

[1] Center for Advanced Materials, Qatar University, Doha 2713, Qatar; mariem.chamakh@qu.edu.qa (M.M.C.); m.alali@qu.edu.qa (M.A.A.A.)

[2] Centre of Polymer Systems, Tomas Bata University in Zlin, Trida T. Bati 5678, 760 01 Zlin, Czech Republic; bazant@utb.cz (P.B.); osicka@utb.cz (J.O.); kuritka@utb.cz (I.K.)

[3] G. W. Woodruff School of Mechanical Engineering, Georgia Institute of Technology, Atlanta, GA 30332, USA; leadenham1@llnl.gov (S.L.); alper.erturk@me.gatech.edu (A.E.)

* Correspondence: mrlik@utb.cz; Tel.:+420-57-603-8027

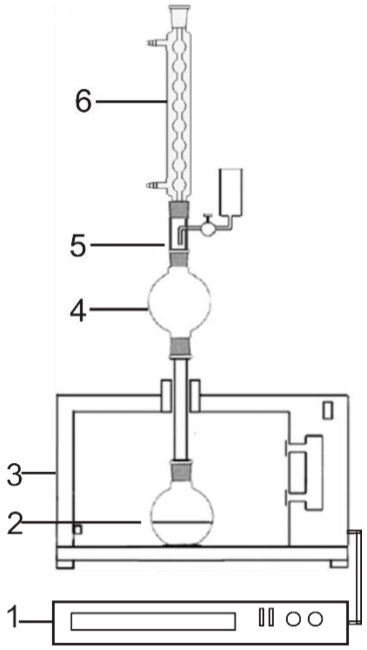

Figure S1. Schematic illustration of the microwave (MW) open vessel apparatus where is 1—external MW source, 2—reaction vessel with temperature control, 3—MW oven, 4—defoamer, 5—dropping system, 6—Allihn condenser.

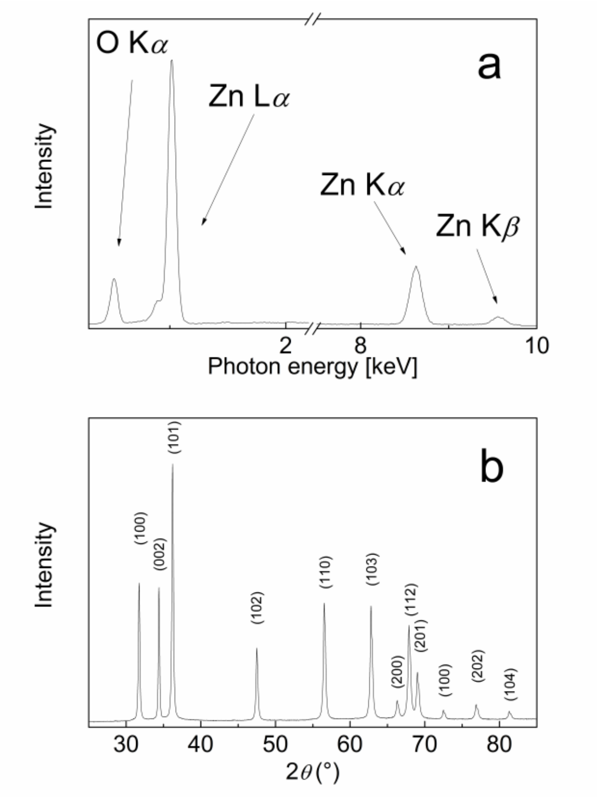

Figure S2. Energy dispersive spectrum (**a**) and X-ray diffraction spectrum (**b**) of conventional ZnO particles.
